# The concordance between colposcopic biopsy and loop electrosurgical excision procedures in patients with known smear cytology and human papillomavirus results

**DOI:** 10.14744/nci.2021.80090

**Published:** 2021-12-31

**Authors:** Sener Gezer, Sumeyye Kanbay Ozturk, Sibel Balci, Izzet Yucesoy

**Affiliations:** 1. Department of Obstetrics and Gynecology, Kocaeli University Faculty of Medicine, Kocaeli, Turkey; 2. Department of Biostatistics and Medical Informatics, Kocaeli University Faculty of Medicine, Kocaeli, Turkey

**Keywords:** Colposcopic biopsy, concordance, loop electrosurgical excision procedure

## Abstract

**Objective:**

The objective of the study was to evaluate the concordance between colposcopic biopsy and loop electrosurgical excision procedure (LEEP) methods to diagnose cervical pre-invasive lesions and cervical cancer, and to calculate the low and high prediction rates of lesions for both methods.

**Methods:**

A total of 241 patients who underwent LEEP after colposcopic biopsy for different indications and also known cervical cytology and human papillomavirus test results were included in the study. Clinical variables such as age, gravida, parity, menopausal status, smoking, endocervical curettage results, and surgical margins were recorded.

**Results:**

The total concordance between colposcopic biopsy and LEEP was 41.9%. The rates of finding a more serious lesion than in colposcopic biopsy with LEEP (underestimation) for negative, Cervical Intraepithelial Neoplasia (CIN) 1, CIN 2, and CIN 3 were calculated as 100%, 12.8%, 14.8%, and 3.9%, respectively. Rates of finding a less serious lesion than detected in colposcopic biopsy with LEEP (overestimation) for CIN 1, CIN 2, and CIN 3, cervical carcinoma were calculated as 56.4%, 33.3%, 3.9%, and 0%, respectively. Underestimation was seen in a total of 28 patients, and overestimation was present in 113 patients. Parity was found to be the only associated factor that affected the final diagnosis for high-grade lesions in univariate logistic regression analysis (odds ratio=1.234, 95% confidence interval: 1.005–1.514).

**Conclusion:**

Discrepancies between colposcopically directed punch biopsy and subsequent histopathologic LEEP findings are common. New methods to reduce the inconsistency between colposcopic biopsy and LEEP are necessary to prevent patients from being under or over treated.

**C**ervical cancer is one of the rare cancers that can be caught pre-invasively with cytologic and high-risk human papillomavirus (HPV) screening and has a chance for early treatment [1]. According to Turkish cancer data from 2014, cervical cancer was the 10^th^ most common cancer among women. Furthermore, most cases (78%) were diagnosed as in situ cancer [2]. Colposcopy is a method of evaluating morphologic changes in the cervix in more detail and increasing the likelihood of biopsy from suspicious areas. If abnormal cytology or HPV positivity is detected, a biopsy taken with colposcopy has an important role in reducing deaths from cervical cancer [3]. Loop electrosurgical excision procedure (LEEP) may be necessary for a more accurate histopathologic diagnosis in such cases; Cervical Intraepithelial Neoplasia (CIN)2/3 is detected in colposcopic biopsy, cytology pathology mismatch, high suspicion of cancer, or when the see-and-treat protocol is to be applied. Although excisional procedures such as cold conization are preferred for lesions with endocervical placement and large-scale tumors, pregnancy complications are more common according to LEEP [4].

Concordance between colposcopic punch biopsy and LEEP conization was reported between 40% and 57% in the literature [5–8]. If a colposcopic biopsy actually considers the existing lesion to be a lower grade lesion, if LEEP is not performed, it will cause a major problem and more recurrence will be detected in these patients. Since LEEP provides the evaluation of the entire transformation zone, it is more reassuring and easy to implement. However, if a lower grade lesion is detected in the LEEP procedure, these patients will be overtreated and face unintended pregnancy complications. The aim of this study was to evaluate the sensitivity, specificity, and concordance rates of colposcopic biopsy and LEEP methods in diagnosing cervical pre-invasive lesions and cervical cancer, and to calculate the low and high prediction rates of lesions for both methods.

## Materials and Methods

This study was conducted through retrospective evaluation of colposcopies performed between 2016 and 2019 in the gynecology department of a university hospital after the approval of the ethics committee (Kocaeli University-GOKAEK 2018/376). The study was carried out in accordance with the Helsinki Declaration “Ethical Principles for Medical Research Involving Human Subjects.” A total of 241 patients who underwent LEEP after colposcopic biopsy for different indications and also had known cervical cytology and HPV test results were included in the study. Adequate colposcopy was considered a clear evaluation of the transformation zone. Colposcopic biopsies and LEEP procedures of all patients were performed at a single center by physicians of at least trainee level in gynecologic oncology. The main indications for LEEP were CIN 2/3 as a result of punch biopsy pathology, high-grade lesion suspicion in colposcopy, non-clear evaluation of transformation zone, cytology biopsy mismatch, and application of the see-and-treat protocol for the lesion. In the case of microinvasion in punch biopsies, LEEP was performed to clearly determine the depth of the invasion. Endocervical curettage was performed in cases where the colposcopic evaluation was inadequate or with the suspicion of endocervical involvement. Pregnant women, patients who had previous cervical surgery or hysterectomy, and patients who had histopathologic results of adenocarcinoma or adenocarcinoma in situ were excluded from the study. All pathology specimens were evaluated by two pathologists experienced in gynecologic oncology. Liquid-based cytology (ThinPrep Pap Test, Hologic, Massachusetts, USA) was used for analyzing cervical smears and the Hybrid Capture 2 (Qiagen, Hilden, GERMANY) was used for high-risk HPV detection. Cervical smear results were classified into the 2014 Bethesda system and histopathology results were also classified in accordance with the recommendations of the American Society for Colposcopy and Cervical Pathology. Clinical variables such as age, gravida, parity, menopausal status, smoking, endocervical curettage results, and surgical margins were recorded.

**Highlight key points**

• Clinicians often encounter discrepancies between colposcopically directed punch biopsy and subsequent histopathological LEEP findings
• The total concordance between colposcopic biopsy and LEEP was 41.9% in this study
• Parity was the only associated factor that affects the exact diagnosis for high-grade lesions.

### Statistical Analysis

All statistical analyses were performed using the IBM SPSS for Windows version 20.0 (SPSS, Chicago, IL, USA) software package. Continuous variables are presented as mean±standard deviation and median (25^th^–75^th^ percentile). Categorical variables are summarized as counts (percentages). The association between two categorical variables was examined using the Chi-square test. Concordance, sensitivity, and specificity analyses were used to compare two the different diagnostic methods. Univariate logistic regression was used to determine the factors affecting the interested variable and the results are expressed as odds ratio (OR) with 95% confidence intervals (CI). All statistical analyses were performed with 5% significance, and a two-sided p<0.05 was considered statistically significant.

## Results

The demographic and clinical data of 241 patients who underwent LEEP after colposcopic biopsy are given in Table 1. The mean age, and median gravida and parity of the patients were 40.5±8.7, 3 (range, 2–4), and 2 (range, 1–3), respectively. Some 80.9% of the patients were premenopausal and 19.1% were postmenopausal. Smoking was present in 63.1% of patients. The most common cytologic abnormality was low-grade squamous intraepithelial lesion (LSIL), and the most common type of HPV was HPV 16. The total concordance between colposcopic biopsy and LEEP in terms of histopathologic diagnosis was calculated as 41.9%. The rates of finding a more serious lesion than colposcopic biopsy with LEEP (underestimation) for negative, CIN 1, CIN 2, and CIN 3 were calculated as 100%, 12.8%, 14.8%, and 3.9%, respectively. The rates of finding a less serious lesion than detected in colposcopic biopsy with LEEP (overestimation) for CIN 1, CIN 2, CIN 3, and cervical carcinoma were calculated as 56.4%, 33.3%, 3.9%, and 0%, respectively (Table 2). Underestimation was seen in a total of 28 patients and overestimation was present in 113 patients. The sensitivity, specificity, and compliance rates for each diagnostic group of colposcopy-directed biopsy are given in Table 3. Parity was found to be the only associated factor that affected the final diagnosis for high-grade lesions in univariate logistic regression analysis (OR=1.234, 95% CI: 1.005–1.514).

**Table 1.** Clinical and demographic data of the patients

	Total, n=241
Age^a^	40.5 (±8.7)
Gravida^b^	3 (2–4)
Parity^b^	2 (1–3)
Menopause status (%)	
Premenopausal	80.9
Postmenopausal	19.1
Smoking (%)	
No	63.1
Yes	36.9
HPV type (%)	
Negative	54.8
16	23.7
18	2.9
31	2.9
33	0.4
35	0.4
52	1.2
56	0.8
58	1.2
Other	11.6
Smear cytology (%)	
Negative	6.2
ASCUS	21.2
LSIL	47.3
HSIL	19.5
ASCH	3.3
AGUS	1.7
Invasive cancer	0.8
Endocervical curettage (ECC) (%)	
None	66.4
Negative	31.1
CIN 1	1.3
CIN 2	0
CIN 3	0.8
Invasive cancer	0.4
Surgical margins (%)	
Negative	85.1
Positive	14.9

a: Mean (Std. deviation); b: Median (25–75. percentile); ASCUS: Atypical squamous cells of undetermined significance; LSIL: Low-grade squamous intraepithelial lesion; HSIL: High-grade squamous intraepithelial lesion; ASCH: Atypical squamous cells cannot exclude HSIL; AGUS: Atypical glandular cells of undetermined significance; CIN: Cervical intraepithelial neoplasia; HPV: Human papillomavirus.

**Table 2.** Comparison of colposcopic biopsy and LEEP

	LEEP (%)						χ^2^(P-value)
Colposcopic biopsy	Negative	CIN 1	CIN 2	CIN 3	Invasive cancer	Total	
Negative	0	0	0	1 (0.4)	0	1 (0.4)	
CIN 1	75 (31.1)	41 (17)	12 (5)	4 (1.7)	1 (0.4)	133 (55.2)	
CIN 2	11 (4.6)	7 (2.9)	28 (11.6)	8 (3.3)	0	54 (22.4)	236.3 (<0.001)
CIN 3	4 (1.7)	5 (2.1)	11 (4.6)	29 (12)	2 (0.8)	51 (21.2)	
Invasive cancer	0	0	0	0	2 (0.8)	2 (0.8)	
Total	90 (37.3)	53 (22)	51 (21.2)	42 (17.4)	5 (2.1)	241 (100)

CIN: Cervical intraepithelial neoplasia; LEEP: Loop electrosurgical excision procedure.

## Discussion

LEEP is preferably used for treating CIN 2–3 lesions because it is safe, cost effective, and practical to perform under local anesthesia in an outpatient setting. However, the alignment between LEEP and punch biopsy may not be enough to satisfy physicians. The present study assessed the frequency of histologic discrepancies between colposcopic biopsies and LEEP specimens in 241 patients. The total concordance rate of this study (41.9%) was similar to previous studies, which reported rates between 40% and 57% [9, 10]. However, Duesing et al. [11] reported higher concordance rates between the two methods. They reported 95.1% concordance for high-grade lesions (CIN 2/3) and 63.2% for low-grade lesions (CIN 1). Better diagnostic efficacy for high-grade lesions (78.5%) than low-grade lesions (33.3%) was found by other authors, and this condition was explained by a greater variability in the pathologic diagnosis of low-grade lesions [12]. Our study found better concordance, sensitivity, and specificity with high-grade lesions (Table 3). Sensitivity rates were reported as 50–75% for low-grade lesions (CIN 1) and 55–90% for high-grade lesions (CIN 2/3) in the literature, whereas specificity varies from 80% for CIN 1 to 96% for CIN 2/3 [12–14].

**Table 3.** Concordance, sensitivity, and specificity rates between colposcopic biopsy and LEEP

	Concordance %	Sensitivity %	Specificity %
Negative	62.2	0	99.3
CIN 1	42.7	47.1	40.3
CIN 2	79.7	54.9	86.3
CIN 3	85.4	69	88.9
Invasive cancer	98.8	66.7	100

CIN: Cervical intraepithelial neoplasia; LEEP: Loop electrosurgical excision procedure.

If a lesion that is detected by colposcopic biopsy indicates lower grade pathology than found in LEEP, this condition involves risks for recurrence and progression. Rates of finding a more serious lesion than colposcopic biopsy with LEEP (underestimation) for negative, CIN 1, CIN 2, and CIN 3 were calculated as 100%, 12.8%, 14.8%, and 3.9%, respectively, in this study. These rates were lower for CIN 1, CIN 2, and CIN 3 than previously reported. In the study by Jung et al. [10], underestimation rates were 75.0%, 24.7%, 23.4%, and 24.2% for biopsy results with normal, CIN 1, CIN 2, and CIN 3, respectively. In a study from Turkey, underestimation rates were similar, 71.42% for negative, 22.91% for CIN 1, 37.03% for CIN 2, and 12.72% for CIN 3 [15]. In another study by Kahramanoglu et al. [16], the underestimation rate for CIN 2+ was 10.5%.

If a lower grade lesion is detected in LEEP, it means that overestimation and overtreatment were made. Rates of finding a less serious lesion than colposcopic biopsy with LEEP (overestimation) for CIN 1, CIN 2, CIN 3, and cervical carcinoma were calculated as 56.4%, 33.3%, 3.9%, and 0%, respectively, in this study. Fourteen to 24% of women with high-grade cervical lesions on colposcopic biopsy have lower grade lesions on LEEP specimens [17–19]. In a study in which the overestimation rate was 16.3% for high-grade lesions, CIN 2 from biopsy was the only statistically significant risk factor for CIN 1 or less in LEEP specimens [20]. This finding was confirmed that of most previous studies which have shown CIN 2 in colposcopic biopsy was the predicting factor of having CIN 1 or less in LEEP specimens [21–23]. In addition, 14.0–29.7% of patients who had previously had biopsy-confirmed CIN had no lesions in LEEP examinations [17, 18, 24]. Possible explanations for this condition are as follows: (1) High-grade lesion was limited to a very small area and completely excised by colposcopic biopsy [25], (2) 6–50% of CIN 2–3 lesions were spontaneously regressed [26], (3) cervical biopsy and/or cone sample may have been misdiagnosed, and (4) LEEP sample was insufficient for diagnosis [18].

Normal or low-grade colposcopic appearance during biopsy may be related to minor LEEP histologic results [27]. Giannella et al. [21] reported that CIN 2 in cervical biopsies and low-grade colposcopic appearance was predictors of minor cone histology. Interestingly, performing LEEP under the guidance of colposcopy may reduce the chances of encountering minor histology with a negative predictive value of 96.9% to predict the low probability of SIL/CIN in the specimen [28]. Performing LEEP under colposcopy improves the ratio of negative margins, minimizes the depth of the excised sample, and improves the accuracy of treatment [29].

Although the number of colposcopic biopsies taken was known to increase the chances of detecting high-grade lesions, it was found to be one of the statistically significant factors for underestimation, along with HPV type and nulliparity [10]. It has even been suggested that taking biopsies from normal appearing cervix might result in more high-grade lesion detection [30].

HPV genotype testing will be another stronger predictor for high-grade lesions. High-risk HPV positivity was found to be associated with CIN2+ cone histology (AOR=0.38, 95% CI: 0.17–0.87) [21]. In addition to HPV positivity, Ryu et al. [18] showed that HPV viral load was a predictive factor for biopsy overestimation without age, Papanicolaou (Pap) test, and punch biopsy grade. Similarly, using HPV viral load and HPV 16 were found as prognostic factors to predict the absence of dysplasia in LEEP specimens [23]. However, another study confirmed that viral load and negative HPV were influencing factors in biopsy overestimation [17]. In women with HPV types 16 and 18 in whom a normal Pap smear was obtained, the probability of developing precancerous cervical lesions was 35 times higher [31]. Furthermore, HPV 16 and 18 positivity were found associated with a higher risk of persistence and progression for CIN [32]. p16 immunohistochemistry was used to decrease the discordance between colposcopic cervical biopsy and LEEP results [22]. This method has been shown again in another study to reduce the frequency of negative LEEP after CIN 2–3 diagnosis of cervical colposcopic biopsies [33].

In the present study, parity was found to be the only associated factor that affected the final diagnosis in univariate logistic regression analysis. In a recently published paper, logistic regression analyses demonstrated that nulliparity, low-grade Pap results, and low-grade colposcopic impressions were significant risk factors for having CIN 1 or less in LEEP specimens [34]. Cesarean section was shown to have no effects, but vaginal delivery was one of the discrepancy factors because of the effect on the squamocolumnar junction. Furthermore, nulliparity was a significant risk factor for CIN in patients who were HIV positive [35].

Another controversial issue is the long-term recurrence risk of patients who have a lower grade lesion in the LEEP specimen. Several follow-up studies have shown that women with underestimated histology did not differ from women with high-grade LEEP histology in relation to disease recurrence; the recurrence rate was between 2% and 9% for these patients [21–23, 36]. However, a positive cone margin in women with CIN2-3 cone histology was a factor influencing the risk of long-term recurrence, as Livasy et al. [37] showed.

The main limitations of this study were its retrospective design that it was performed with few patients in single center, the lack of some clinical variables such as the size of cervical lesions of the LEEP specimens, and the absence of long-term follow-up and recurrence rates. Furthermore, we did not gather information about the number of biopsies taken per patient; this should be subject to further research.

### Conclusion

The total concordance between colposcopic biopsy and LEEP in terms of histopathologic diagnosis was similar to the literature. Furthermore, we were not able to identify any factors other than parity that could affect the final diagnosis. The discrepancy between colposcopically directed punch biopsy and subsequent histopathologic LEEP findings is common and it is not easy for physicians. HPV genotyping and P16 immunohistochemistry staining and performing LEEP cone biopsy under colposcopic observation may be alternative methods that could be used to overcome this problem. Applying a standard biopsy procedure can reduce the variability between practitioners, especially in a university service. There is also a need to raise awareness of physicians who inform patients about the results.
